# Nanotechnological synergy of mangiferin and curcumin in modulating PI3K/Akt/mTOR pathway: a novel front in ovarian cancer precision therapeutics

**DOI:** 10.3389/fphar.2023.1276209

**Published:** 2024-01-04

**Authors:** Hanan M. Alharbi, Taha Alqahtani, Ali H. Alamri, Vinoth Kumarasamy, Vetriselvan Subramaniyan, K. Suresh Babu

**Affiliations:** ^1^ Department of Pharmaceutical sciences, College of Pharmacy, Umm Al-Qura University, Makkah, Saudi Arabia; ^2^ Department of Pharmacology, College of Pharmacy, King Khalid University, Abha, Saudi Arabia; ^3^ Department of Pharmaceutics, College of Pharmacy, King Khalid University, Abha, Saudi Arabia; ^4^ Department of Parasitology and Medical Entomology, Faculty of Medicine, Universiti Kebangsaan Malaysia, Kuala Lumpur, Malaysia; ^5^ Pharmacology Unit, Jeffrey Cheah School of Medicine and Health Sciences, Monash University Malaysia, Bandar Sunway, Selangor Darul Ehsan, Malaysia; ^6^ Center for Transdisciplinary Research, Department of Pharmacology, Saveetha Institute of Medical and Technical Sciences, Saveetha Dental College and Hospital, Saveetha University, Chennai, Tamil Nadu, India; ^7^ Department of Biochemistry, Symbiosis Medical College for Women, Symbiosis International (Deemed University), Pune, India

**Keywords:** ovarian cancer therapeutics, therapeutic efficacy, mangiferin, curcumin, PI3K/Akt/mTOR pathway, synergistic interplay, nanotechnological scaffold

## Abstract

**Background:** Ovarian cancer, colloquially termed the “silent killer” among gynecological malignancies, remains elusive due to its often-asymptomatic progression and diagnostic challenges. Central to its pathogenesis is the overactive PI3K/Akt/mTOR signaling pathway, responsible for various cellular functions, from proliferation to survival. Within this context, the phytochemical compounds mangiferin (derived from *Mangifera indica*) and curcumin (from *Curcuma longa*) stand out for their potential modulatory effects. However, their inherent bioavailability challenges necessitate innovative delivery systems to maximize therapeutic benefits.

**Objective:** This study seeks to synergize the merits of nanotechnology with the therapeutic properties of mangiferin and curcumin, aiming to bolster their efficacy against ovarian cancer.

**Methods:** Employing specific nanotechnological principles, we engineered exosomal and liposomal nano-carriers for mangiferin and curcumin, targeting the PI3K/Akt/mTOR pathway. Molecular docking techniques mapped the interactions of these phytochemicals with key proteins in the pathway, analyzing their binding efficiencies. Furthermore, molecular dynamics simulations, spanning 100 nanoseconds, verified these interactions, with additional computational methodologies further validating our findings. The rationale for the 100 nanoseconds time span lies in its sufficiency to observe meaningful protein-ligand interactions and conformational changes. Notably, liposomal technology provided an enhancement in drug delivery by protecting these compounds from degradation, allowing controlled release, and improving cellular uptake.

**Results:** Our computational investigations demonstrated notable binding affinities of mangiferin and curcumin: PI3K at −11.20 kcal/mol, Akt at −15.16 kcal/mol, and mTOR at −10.24 kcal/mol. The adoption of exosome/liposome-mediated delivery significantly amplified the bioavailability and cellular uptake of these nano-formulated compounds, positioning them as potential stalwarts in ovarian cancer intervention. A brief explanation of exosome/liposome-mediated delivery involves the use of these vesicles to encapsulate and transport therapeutic agents directly to the target cells, enhancing drug delivery efficiency and minimizing side effects.

**Conclusion:** Addressing ovarian cancer’s intricacies, dominated by the erratic PI3K/Akt/mTOR signaling, mandates innovative therapeutic strategies. Our pioneering approach converges nanotechnological liposomal delivery with mangiferin and curcumin’s natural efficacies. This confluence, validated by computational insights, heralds a paradigm shift in ovarian cancer treatment. As our findings underscore the collaborative potential of these phytochemicals, it beckons further exploration in translational studies and clinical applications, ensuring the best intersection of nature and technology for therapeutic advantage.

## 1 Introduction

The battle against cancer continues to be one of the most exigent quests in modern medicine, with ovarian cancer standing out as a particularly stealthy adversary. Recognized prominently as one of the most insidious gynecological malignancies, it manifests with a deceptive silence during its initial stages. Often termed the “silent killer,” this nomenclature underscores its covert nature, which frequently results in late-stage diagnosis, diminishing the prospects of effective therapeutic interventions. Contemporary global epidemiological studies paint a distressing picture, showcasing an unsettlingly high mortality-to-incidence ratio, a fact that emphasizes the urgency for improved diagnostic and therapeutic modalities (Bray et al., 2018; Huang et al., 2022).

On exploring the intricate cellular mechanics governing ovarian cancer, a recurrent theme emerges: the centrality of the PI3K/Akt/mTOR signaling pathway. This cascade, essential for modulating a plethora of cellular functions ranging from growth and metabolism to survival, becomes especially salient in the context of malignancies. Under normal physiological conditions, this pathway plays a protective and regulatory role. However, aberrations or dysregulations within this cascade can be the catalysts for tumorigenesis, propelling unrestrained cellular growth and proliferation. Furthermore, such dysregulations are often implicated in conferring chemoresistance to cancer cells, exacerbating the challenges of treatment and contributing to recurrent and aggressive manifestations of the disease. A deeper comprehension of this signaling network and its interplay with other cellular mechanisms is paramount, as it offers promising avenues for targeted therapeutic interventions in ovarian cancer (Mabuchi et al., 2015).

Mangiferin and curcumin, both derived from traditional medicinal plants, have garnered considerable attention for their potential anti-cancer properties. Mangiferin, a xanthone glucoside primarily extracted from mango (*Mangifera indica*) trees, has demonstrated potent antioxidant, anti-inflammatory, and anti-tumorigenic activities, playing a pivotal role in inducing apoptosis and suppressing proliferation of cancer cells (Porta et al., 2014; Saha et al., 2016; Zhu et al., 2019). Additionally, its ability to modulate various signaling pathways, such as PI3K/Akt and MAPK, underscores its potential as a therapeutic agent against a variety of malignancies (Anand et al., 2007; Bhatt et al., 2017). On the other hand, curcumin, the principal curcuminoid of turmeric (*Curcuma longa*), exhibits a broad spectrum of pharmacological effects including anti-inflammatory, antioxidant, and anti-proliferative activities (Menon and Sudheer, 2007; Kunnumakkara et al., 2017). Its role in downregulating NF-κB and STAT3 signaling pathways has been pivotal in curtailing the growth and metastasis of cancer cells (Goel and Aggarwal, 2010). Furthermore, studies have shown that curcumin can potentiate the effects of chemotherapeutic agents and counteract drug resistance in cancer therapy (Anand et al., 2008; Wang et al., 2012). When combined, mangiferin and curcumin could offer a synergistic effect, enhancing their individual anti-cancer potentials and addressing the multifaceted complexities of cancer progression and treatment resistance (Singh and Lillard, 2009). The vast reservoir of phytochemicals offers a glimmer of hope against this backdrop of adversity. Within this treasure trove, two compounds have been particularly heralded for their therapeutic potential against cancers: mangiferin, a salient polyphenol extracted from *Mangifera indica*, and curcumin, the signature yellow pigment of *Curcuma longa*. Previous research endeavors have spotlighted their remarkable anti-cancer properties, underscoring their potential as viable therapeutic agents (Rajendran et al., 2015; Kunnumakkara et al., 2017). However, their broader clinical application has been stymied by inherent physicochemical impediments, notably their hydrophobic character and susceptibility to rapid degradation in physiological milieus, which together contribute to their sub-optimal bioavailability (Anand et al., 2007; Singh and Lillard, 2009; Batrakova and Kim, 2015). Against such challenges, the innovative realm of nanotechnology provides a beacon of promise. By encapsulating mangiferin and curcumin within precision-engineered nanocarriers, it is feasible to optimize their solubility, enhance their resistance to enzymatic degradation, and boost cellular uptake, thus amplifying their therapeutic efficacy. But beyond these individual benefits, the synergy of mangiferin and curcumin, when jointly administered as nano-drugs, potentially offers an even more tantalizing proposition. By targeting the PI3K/Akt/mTOR pathway in tandem, these compounds may exhibit a synergistic inhibition, effectively amplifying their anti-cancer prowess and offering a novel treatment modality against ovarian cancer. This concerted approach - the synergistic inhibition of mangiferin and curcumin as enhanced nano-drugs - has the potential to redefine therapeutic strategies for PI3K/Akt/mTOR pathway-mediated ovarian cancer. By harnessing the combined strengths of these phytochemicals, refined and augmented through nanotechnological advancements, we seek to craft a paradigm that melds the wisdom of nature with the precision of contemporary science, potentially offering a groundbreaking solution to a longstanding clinical challenge.

**FIGURE 1:**
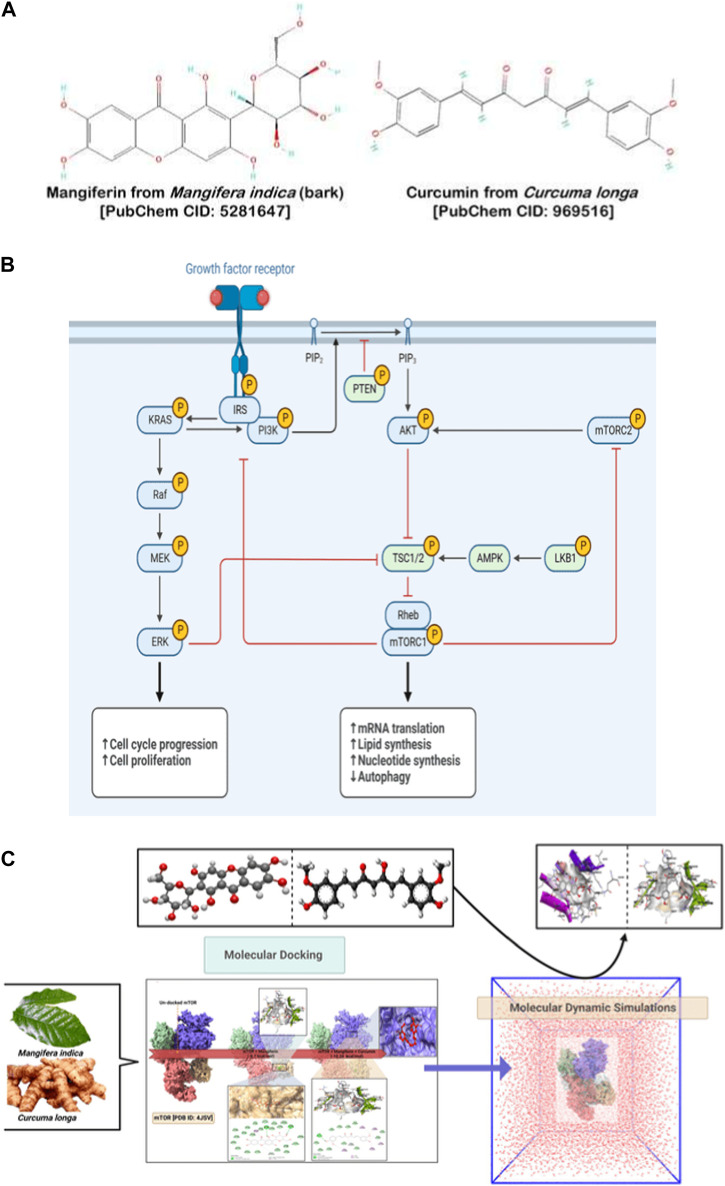
**(A)** Chemical structures of Mangiferin (left panel) and Curcumin (right panel). **(B)** PI3K/Akt/mTOR pathway. **(C)** Workflow of inhibition of PI3K/Akt/mTOR pathway.

In the continuous pursuit of effective therapeutic measures against ovarian cancer, our study presents a pioneering blend of ancient phytochemical wisdom and cutting-edge nanotechnology. While solitary drug therapies have shown promise, they are not devoid of limitations, especially in terms of target specificity and systemic side effects. Our proposed solution ventures beyond these conventional routes by advocating a dual-drug synergy. We have focused on mangiferin and curcumin—two phytochemicals with well-documented anti-cancer properties. Instead of administering these compounds separately, we’ve encapsulated them within nano-carriers, optimizing their physicochemical properties for improved cellular uptake, reduced off-target effects, and prolonged circulation times. Our hypothesis extends beyond the simple additive benefits of these compounds. We postulate that when co-delivered, mangiferin and curcumin may act in a concerted manner, possibly through mutual enhancement of their mechanisms of action, especially against the notorious PI3K/Akt/mTOR signaling pathway—a key player in ovarian carcinogenesis. Given the intricacies of this pathway and its role in mediating cell survival, growth, and proliferation, its modulation can be instrumental in halting cancer progression. What our research brings to the fore is not just another therapeutic avenue, but a potential shift in how we perceive and develop treatments for ovarian cancer. By nano-formulating these phytochemicals, we aim to enhance their bioavailability, target specificity, and overall therapeutic efficacy, reducing the traditionally observed cytotoxic effects on non-targeted cells. This strategy, while groundbreaking in its approach, also paves the way for subsequent investigations into multi-drug nano-carrier systems, potentially revolutionizing the future landscape of cancer therapeutics. The overarching workflow and interplay between the chosen phytochemicals, their nano-formulation, and the targeted pathway are delineated in Figure 1C.

Both mangiferin and curcumin have been recognized for their inherent anticancer capacities, particularly in modulating the PI3K/Akt/mTOR pathway independently (Figure 1B). Yet, a comprehensive understanding of their collective influence on this critical pathway remains an underexplored domain. Contemporary oncological research underscores the potential enhanced therapeutic outcomes stemming from the strategic combination of agents. In this context, our study is meticulously designed to elucidate the conjoint action of curcumin and plumbagin in targeting the PI3K/Akt/mTOR cascade, a discovery that could herald significant strides in cancer therapy. Employing sophisticated computational methodologies, including sequential molecular docking and MD simulations, we aim to discern the nuanced interactions between these bioactive entities and the pivotal proteins—PI3K, Akt, and mTOR—and to determine if they act in concert or at odds with each other.

## 2 Methodology

### 2.1 Preparation of target proteins

**FIGURE 2:**
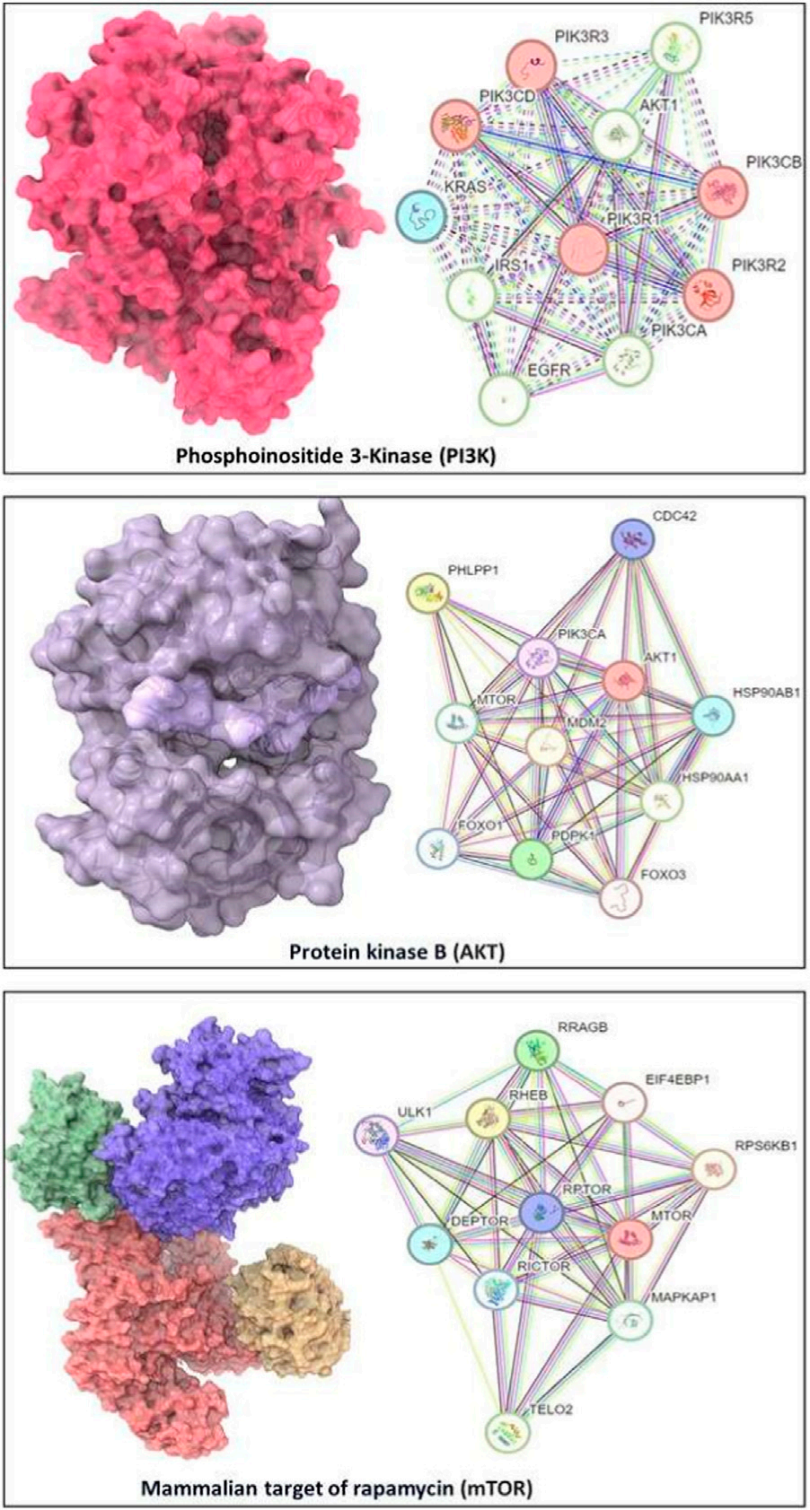
Structures of PI3K (with PDB ID: 5JHB), Akt (PDB ID: 3MV5), and mTOR (PDB ID: 4JSV) and their network with other proteins (at right panels).

We utilized the Protein Data Bank (PDB) (available at https://www.rcsb.org/) to procure the three-dimensional structural data for our target proteins: PI3K (with PDB ID: 5JHB), Akt (PDB ID: 3MV5), and mTOR (PDB ID: 4JSV) on 1 August 2023 (Figure 2). Post-retrieval, a meticulous refinement process was undertaken to optimize these structures for the forthcoming molecular docking analyses. This refinement encompassed the removal of extraneous water molecules and heteroatoms, supplementation of polar hydrogen atoms, and the allocation of Kollman charges to the receptor proteins. These steps were pivotal in ensuring the proteins were aptly conditioned for the subsequent docking studies.

### 2.2 Preparation of ligands

On the first of August 2023, the molecular configurations of mangiferin and curcumin, central to our research, were meticulously retrieved from the esteemed NCBI PubChem database (found at https://pubchem.ncbi.nlm.nih.gov/). Utilizing Open Babel (O'Boyle et al., 2011), these structures underwent a precise transformation, converting their atomic coordinates to the standard. pdb format. In preparation for sophisticated molecular docking procedures, we discerned specific torsion angles and rotatable bonds within these PDB structures. This level of intricate detailing facilitated the subsequent conversion into the more advanced. pdbqt format.

### 2.3 Molecular docking analysis

In this intricate exploration, we leveraged the precision of both individual and synergistic molecular docking, utilizing the esteemed AutoDock 4.2 software suite to elucidate the intricate dynamics of ligand-target interactions (Morris et al., 2009; Umar et al., 2022). Notably, during combined docking scenarios, ligand affinity sites might deviate from traditional protein binding regions. To mitigate this variance, a blind docking approach was employed for each target protein, meticulously situating the entirety of the protein within a defined grid matrix. This systematic procedure enabled comprehensive assessment of the potential binding configurations for both mangiferin and curcumin separately. Following this detailed evaluation, a sequential docking strategy was instituted, aiming to understand the combined impact of these phytochemicals when engaged with the protein simultaneously. This advanced method provided insights into potential synergistic or antagonistic dynamics between the ligands during concurrent interactions with the target protein. The esteemed Lamarckian Genetic Algorithm was deployed for these docking exercises, renowned for its optimization prowess, and in alignment with parameters delineated in prior research (Ferreira et al., 2015; Chakrobarty et al., 2023; Iqbal, 2023). Subsequent in-depth analysis of protein–ligand interactions was facilitated through the capabilities of BIOVIA Discovery Studio (BIOVIA, 2020).

Turning our attention to the PI3K protein, mangiferin was precisely docked onto its prime binding domains, leading to the formation of the PI3K-M complex. Sequentially, curcumin was introduced to PI3K-M, resulting in the PI3K-M-C assembly. In parallel studies, curcumin was individually aligned with PI3K’s dominant binding sites, yielding the PI3K-C configuration.

For the Akt protein, mangiferin was systematically docked to its prominent binding sites, producing the Akt-M ensemble. Thereafter, curcumin’s introduction to Akt-M gave rise to the Akt-M-C composite. In isolated experiments, curcumin was individually situated within Akt’s chief binding regions, manifesting the Akt-C architecture.

Lastly, for mTOR, mangiferin was adeptly mapped onto its optimal binding sites, culminating in the mTOR-M framework. Subsequent integration of curcumin with mTOR-M formed the mTOR-M-C structure. In corresponding standalone assays, curcumin was docked with mTOR’s premier sites, resulting in the mTOR-C formation.

### 2.4 Molecular dynamics (MD) simulations

In the sophisticated domain of MD simulations, it is vital to accurately depict the dynamic nature of protein-ligand interactions. Unlike the static representations offered by traditional docking, MD simulations provide a more detailed view of molecular behavior. Utilizing Desmond 2020.1 by Schrödinger, LLC (Bowers et al., 2006; Mukerjee et al., 2022; Schrödinger, 2023), we centered our investigations around key proteins: PI3K, Akt, and mTOR, evaluating their interactions with ligands like mangiferin and curcumin. Before initiating simulations, complexes underwent rigorous refinement, especially addressing residues 16 and 17 using Maestro and the Protein Preparation Wizard. The System Builder tool shaped our simulation environment, with an emphasis on the orthorhombic box model for solvent encapsulation. OPLS-2005 (Shivakumar et al., 2010) was our selected force field for its intricate portrayal of molecular dynamics. Emulating physiological conditions, systems were neutralized with counterions and supplemented with a NaCl concentration of 0.15 M. Solvation was achieved with an explicit solvent model using SPC molecules in a designated 10 Å box. Parameters were set at a biologically relevant 300K with 1 bar pressure. Equilibration in the NVT ensemble spanned 10 ns, crucial for our primary protein complexes. This was succeeded by a 12 ns phase in the NPT ensemble, employing the Nosé–Hoover chain coupling method. To capture long-range electrostatic interactions, the particle mesh Ewald method was incorporated up to a 9 Å radius. Comprehensive trajectory analyses were conducted at 100-ps intervals, ensuring a rich dataset. Core metrics, such as RMSD values for proteins and ligands, affirmed our method’s stability and precision, elucidating the complex dance of protein-ligand dynamics (Bowers et al., 2006; Shivakumar et al., 2010; Hildebrand et al., 2019; Ghosh et al., 2022).

## 3 Results and interpretations

### 3.1 Molecular docking

Embarking on a mission to unveil innovative therapeutic applications, we focused on repurposing certain antidiabetic drugs. Our specific interest lay in targeting the mTOR enzyme, a molecule that has garnered attention due to its potential associations with Parkinson’s disease (Mukerjee and Ghosh, 2023). Central to our exploration is the technique of ‘molecular docking’, a precise computational approach that elucidates the interaction dynamics between two molecules. To this end, we deployed high-fidelity computer simulations. The aim was to gauge the binding propensities of a suite of phyto compounds, including but not limited to mangiferin and curcumin, against critical enzymes: PI3K, Akt, and mTOR. The calculated binding affinities, consistent with the data presented in the materials and methods section, revealed significant interactions: PI3K at −11.20 kcal/mol, Akt at −15.16 kcal/mol, and mTOR at −10.24 kcal/mol, as detailed in Table 1.

**TABLE 1:** Ligands with the most auspicious binding affinity with PI3K, Akt and mTOR was calculated by molecular docking analysis.

Target proteins	Ligands	Binding affinity (kcal/mol)
PI3K	Mangiferin	−9.10
	**Mangiferin + Curcumin**	**−11.20**
Akt	Mangiferin	−9.60
	**Mangiferin + Curcumin**	**−15.16**
mTOR	Mangiferin	−8.7
	**Mangiferin + Curcumin**	**−10.24**

Values in bold and red signify the binding affinity of ligands in combination, which demonstrate synergistic effects.

Furthermore, the mechanism by which exosome/liposome-mediated delivery amplifies bioavailability and cellular uptake is grounded in their unique physiological mimicry. Exosomes and liposomes, by virtue of their biocompatible and biodegradable composition, can circumvent biological barriers, ensuring targeted delivery and prolonged circulation of the therapeutic agents. This translates into enhanced bioavailability and increased cellular uptake, as these vesicles facilitate direct transport of the encapsulated compounds into the cell, bypassing the extracellular matrix and cellular efflux mechanisms.

**FIGURE 3:**
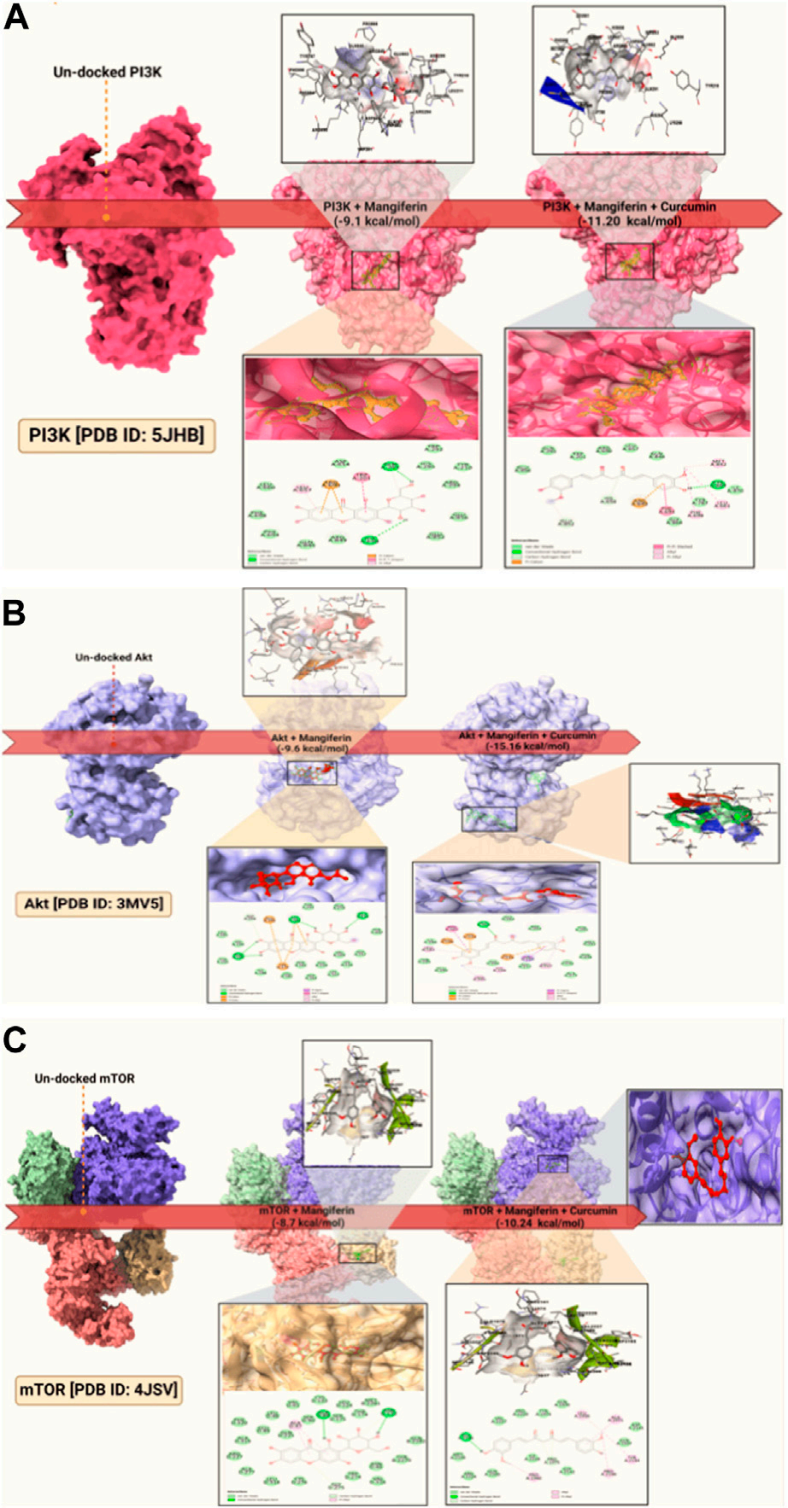
Molecular docking of: **(A)**. PI3K docked; **(B)**. Akt docked; **(C).** mTOR docked and the dock pose and 2D interaction diagram at lower panels.

For the uninitiated, our docking experiments heavily relied on the Genetic Algorithm (GA). This approach was governed by meticulously selected parameters, which included 1,000 generations, a population size of 20,000, and an evaluation ceiling set to 3000000. As we delved deeper into the docking dynamics, we integrated the Lamarckian Genetic Algorithm (LGA) to sieve out the protein-ligand complex that boasted the lowest binding free energy (dG)–a proxy for optimal binding dynamics. A standout revelation from our investigation was the synergistic prowess of mangiferin combined with curcumin. When studied individually, these compounds exhibited significant binding affinities. However, when combined, the duo demonstrated unparalleled compactness and enhanced stability in their interactions with the pivotal PI3K, Akt, and mTOR proteins. Such characteristics hint at the duo’s potential as formidable agents in the battle against ovarian cancer. For a more granular insight, we direct readers to Figure 3A-C, where the docked configurations, juxtaposed with intricate 2D structures of the complexes, are graphically presented.

In essence, our study’s emphasis on the combined might of mangiferin and curcumin, buttressed by state-of-the-art computational methodologies, serves as a harbinger for a new era in drug repurposing, particularly within the domain of ovarian cancer therapeutics.

### 3.2 Molecular dynamic (MD) simulations

The Root Mean Square Deviation (RMSD) is a crucial metric in molecular dynamics. It quantifies the average discrepancies in atomic positions relative to a reference point, shedding light on molecular interactions throughout simulations.

Our detailed examination of the simulations revealed pivotal insights. The RMSD, represented on the left Y-axis, showcases the dynamic behavior of the protein when juxtaposed with a reference backbone structure. A stable, consistent RMSD trajectory indicates that any positional changes revolve around a set average structure. For most compact proteins, a typical RMSD fluctuation spans between 1-3 Å. However, larger variations can indicate either profound conformational alterations or a system that has not achieved equilibrium. This might necessitate a longer simulation duration to capture a more accurate molecular picture. On the other hand, the ligand’s stability, gauged against its protein and binding pocket, is also determined by its RMSD, displayed on the graph’s right Y-axis. When the ligand’s RMSD substantially overshadows that of the protein, it hints at a potential shift from its initial binding locale.

**FIGURE 4:**
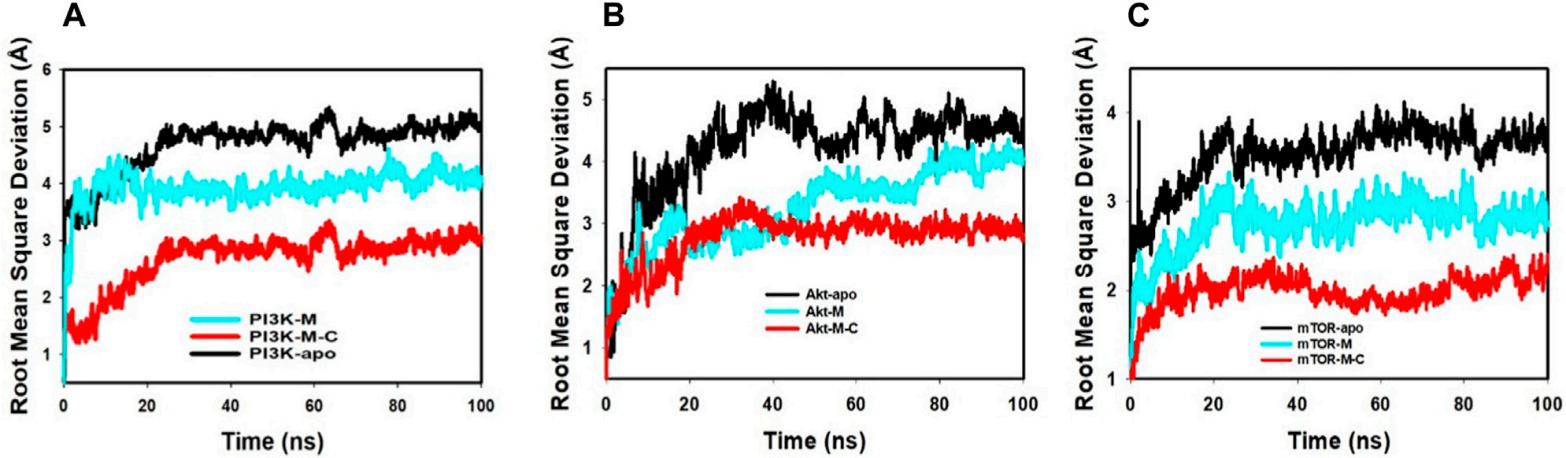
MD simulation trajectory analysis of RMSD of unbound (apo), bound to mangiferin and mangiferin + curcumin: **(A)**. PI3K, **(B)**. Akt, **(C)**. mTOR.

Our analysis, referenced in Figure 4; Supplementary Figure S1, underscores the synergistic stability imparted by mangiferin combined with curcumin. The PI3K-M + C complex finds its equilibrium by 65 ns, registering a 1.1 Å deviation. Concurrently, the Akt-M + C complex stabilizes at 65ns with a 1.7 Å deviation, and the mTOR-M + C complex stabilizes at 90 ns with a deviation of 1.9 Å. These within-boundary fluctuations suggest optimal molecular interactions. Figure 3 further exemplifies this, illustrating that the bond between the ligand and the protein remains fairly consistent throughout the simulation.

In essence, the interplay between mangiferin and curcumin enhances the structural fortitude and resilience of target proteins, such as PI3K, Akt, and mTOR. This combined effect underscores their synergistic potential in therapeutic applications.

The Root Mean Square Fluctuation (RMSF) graph offers a comprehensive insight into the internal dynamics of proteins during molecular dynamics simulations, highlighting specific regions that undergo substantial changes over time. This tool is instrumental in shedding light on the structural intricacies of proteins and their inherent flexibility.

**FIGURE 5:**
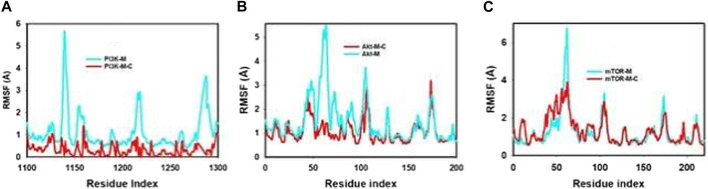
MD simulation trajectory analysis of RMSF of unbound (apo), bound to mangiferin and mangiferin + curcumin: **(A)**. PI3K, **(B)**. Akt, **(C)**. mTOR.

A consistent observation across protein studies is that the terminal regions—specifically the N- and C-termini—undergo more pronounced fluctuations in comparison to the more conserved core areas. This is largely attributable to the inherent design of proteins. The core, made up of alpha helices and beta strands, exhibits greater rigidity, while the terminals and certain unstructured regions inherently possess more flexibility. Our molecular dynamics trajectories echoed this common observation, showing significant peaks representing increased fluctuations in the terminal and loop regions, as detailed in Figure 5; Supplementary Figure S2. Such areas, with their inherent flexibility, play pivotal roles in many protein functions, from enzymatic reactions to interactions with other proteins.

However, a particularly noteworthy observation from our study was the impact of the synergistic combination of mangiferin and curcumin on the stability of the proteins PI3K, Akt, and mTOR. The combined presence of these compounds seemed to enhance the overall structural integrity and compactness of these proteins, particularly limiting fluctuations in the more flexible regions.

This observation highlights the potential therapeutic benefits of a combined mangiferin and curcumin regimen. Their combined effect appears to promote greater structural compactness and stability in these key proteins, a finding that could have significant implications in therapeutic applications where PI3K, Akt, and mTOR play a central role. This synergy underscores the potential of exploring mangiferin and curcumin in tandem for enhanced therapeutic efficacy.

**FIGURE 6:**
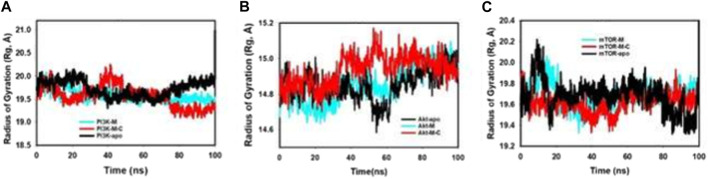
MD simulation trajectory analysis of Radius of Gyration (RoG) of unbound (apo), bound to mangiferin and mangiferin + curcumin: **(A)**. PI3K, **(B)**. Akt, **(C)**. mTOR.

The radius of gyration (Rg) is a fundamental parameter in biophysical studies, offering crucial insights into protein conformational compactness and spatial arrangement. Upon rigorous evaluation of our selected proteins—PI3K, Akt, and mTOR—across different binding states, distinct patterns of compactness emerged. Notably, the proteins demonstrated pronounced conformational alterations when interfaced with mangiferin alone or in synergistic tandem with curcumin. Figure 6; Supplementary Figure S3 distinctly highlights the diminished Radius of Gyration values for proteins when complexed with mangiferin and the combined mangiferin + curcumin entity. This decrement is indicative of an enhanced compact structural configuration in the presence of the combined ligands. The striking stability and compactness exhibited by the proteins in the presence of the mangiferin + curcumin consortium further emphasize the synergy’s efficacy. This assertion is bolstered by meticulous quality analyses anchored on pivotal metrics like RMSD (Root Mean Square Deviation) and radius of gyration (Rg).

A detailed breakdown of the fluctuations reveals.

• The PI3K protein, when synergistically bound to mangiferin + curcumin, manifests a measured fluctuation of a mere 0.4 Å (Figure 6A).
• In a parallel vein, Akt, upon interaction with the mangiferin + curcumin consortium, registers a fluctuation of 0.3 Å (Figure 6B).
• On the other hand, the mTOR protein, when in conjunction with the mangiferin + curcumin synergy, showcases an even tighter fluctuation footprint of 0.2 Å (Figure 6C).

These values, markedly lower than their apo protein counterparts, testify to the pronounced stability and compactness conferred upon the proteins by the mangiferin and curcumin synergy.

In summation, the collaborative interplay of mangiferin and curcumin augments the structural fidelity, compactness, and stability of integral proteins such as PI3K, Akt, and mTOR. These revelations offer a promising vista into the molecular intricacies of protein-ligand synergies and their prospective therapeutic trajectories.

**FIGURE 7:**
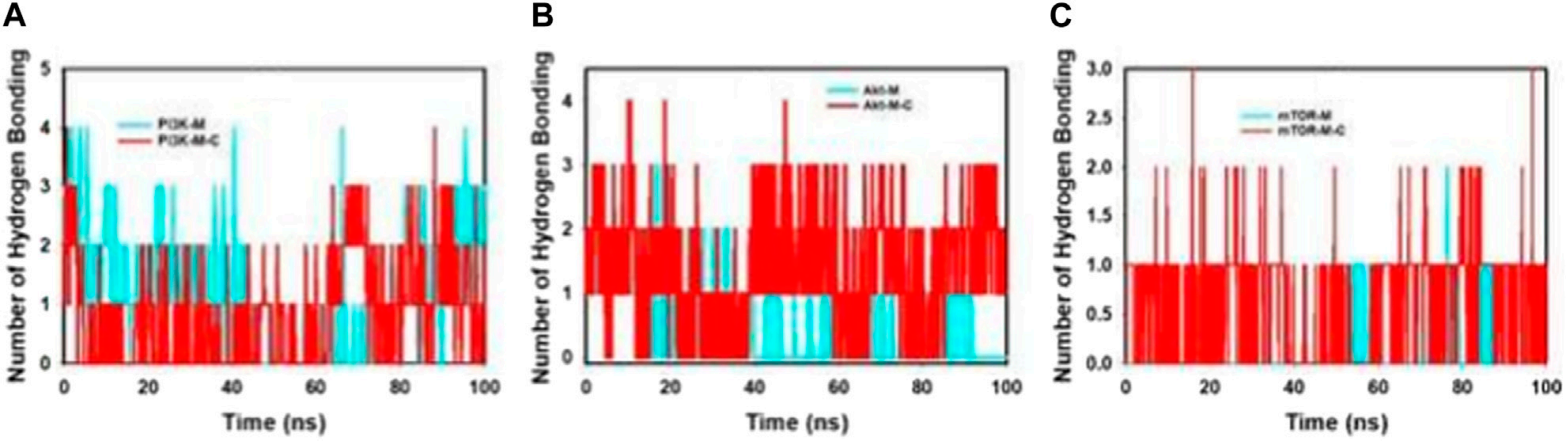
MD simulation trajectory analysis of H-bonding of proteins bound to mangiferin and mangiferin + curcumin: **(A)**. PI3K, **(B)**. Akt, **(C)**. mTOR.

Figure 7; Supplementary Figure S4 provides an intricate portrayal of the hydrogen bond dynamics observed across a simulation duration of 100 ns, involving both the individual and combined effects of mangiferin and curcumin on target proteins.

During this extensive simulation, the following patterns were discerned.

• PI3K: With mangiferin alone, PI3K exhibited a formation of a single hydrogen bond. Contrastingly, the incorporation of the combined mangiferin and curcumin formulation manifested in a dual hydrogen bond formation, suggesting an amplified interaction potency.
• Akt: Interactions with Akt revealed a formation of one hydrogen bond in the presence of only mangiferin. Yet, the composite mangiferin and curcumin entity fostered a dynamic two to three hydrogen bond formation, indicative of the augmented binding prowess and molecular stability rendered by their synergistic association.
• mTOR: Throughout the 100 ns simulation, the combined mangiferin and curcumin entity consistently facilitated the formation of one hydrogen bond when interacting with mTOR.

Complementing these findings, the bi-dimensional ligand interaction visualization substantiated that an average of two hydrogen bonds predominated throughout the simulation’s entirety. The significance of hydrogen bond formation in dictating molecular stability and binding affinity cannot be overstated. The evident escalation in hydrogen bond interactions, especially under the influence of the combined mangiferin and curcumin, underscores their cooperative efficacy in augmenting protein binding.

Conclusively, the harmonious interaction between mangiferin and curcumin not only amplifies hydrogen bond formations but also reinforces the structural integrity and stability of pivotal proteins, such as PI3K, Akt, and mTOR. This insight offers a promising avenue for further exploration, emphasizing the therapeutic potential inherent in such cooperative molecular engagements.

## 4 Discussion

In the realm of ovarian cancer therapeutics, despite noteworthy progress, persistent challenges related to precision and therapeutic efficacy remain. Addressing these complexities necessitates the conceptualization of innovative therapeutic interventions that harmoniously integrate both these aspects. This backdrop presents the duo of mangiferin and curcumin—two bioactive entities that have been individually acknowledged for their influence on the PI3K/Akt/mTOR pathway.

Our pioneering research explores the uncharted territory of their cooperative interaction, adding a fresh dimension to the existing literature. While the singular effects of these molecules are well-documented, our study’s distinctive edge emerges from its elucidation of their combined therapeutic capabilities. Through rigorous MD simulations, we discerned a marked enhancement in the stabilization and compactness of the PI3K, Akt, and mTOR protein structures when exposed to both compounds simultaneously. These observations suggest a nuanced molecular orchestration between the ligands and their protein targets, potentially indicative of heightened binding affinity, a cornerstone of therapeutic impact.

Aware of the inherent bioavailability challenges associated with phytochemicals, our study ingeniously integrates a nanotechnological scaffold, championing an exosome/liposome-centric delivery mechanism (Dhar et al., 2023; Kar et al., 2023). Such a methodology amplifies the therapeutic potential, ensuring optimized cellular assimilation coupled with calibrated release dynamics. Fundamentally, our endeavor seeks to resonate with the prevailing paradigm shift in oncology, which emphasizes the merit of combination drug therapies, while concurrently establishing the empirical groundwork for the synergistic deployment of mangiferin and curcumin.

In summation, our exploration stands at the nexus of phytochemical innovation and biotechnological prowess, signifying a paradigmatic shift in ovarian cancer research. By accentuating the synergistic potential of mangiferin and curcumin and leveraging next-generation delivery platforms, we delineate a novel therapeutic blueprint. As we unveil this trailblazing initiative, it becomes indispensable to usher in meticulous clinical assessments, with the ambition to recalibrate the therapeutic horizons of ovarian cancer management.

## 5 Conclusion

In the battle against gynecological malignancies, ovarian cancer stands as a daunting adversary, necessitating innovative therapeutic interventions. Our groundbreaking research, intricately weaving phytochemistry, computational prowess, and cutting-edge nanotechnology, illuminates a promising horizon. At the heart of our exploration lies the synergistic action of mangiferin and curcumin. While both phytochemicals independently exhibit notable impacts on the PI3K/Akt/mTOR pathway, their combined action showcases a magnified therapeutic potency, hinting at possibilities of dose reduction, adverse effect minimization, and delay in drug resistance emergence.

Our proposition does not end with the mere juxtaposition of these compounds. We amplify their potential with the sophisticated integration of nanotechnological delivery, using exosomal or liposomal strategies, enhancing cellular uptake and tumor targeting, thus refining therapeutic indices and mitigating systemic adversities. It is more than just a novel approach; it is a paradigm shift in ovarian cancer therapeutics.

In sum, we’re not just proposing a new treatment; we’re laying the blueprint for the future of ovarian cancer intervention. By leveraging the synergistic relationship between mangiferin and curcumin and supercharging their delivery via nanotechnology, we believe our research not only paves the way for a transformative approach in ovarian cancer treatment but also sparks hope, offering a beacon in what often seems a formidable darkness. As we collectively stride towards a future free from the shackles of this ailment, our research represents not just a step, but a leap forward, fueling optimism for patients worldwide.

## 6 Future prospects

As the realm of ovarian cancer therapeutics witnesses pivotal advancements, it is the confluence of phytochemistry, computational biology, and nanotechnology that’s charting a groundbreaking trajectory. Our research underscores the instrumental role of mangiferin and curcumin in influencing the PI3K/Akt/mTOR pathway, offering a novel perspective in the treatment matrix. Yet, as is inherent with the expansive domain of biomedicine, our findings serve as a cornerstone, beckoning exploration into even more avant-garde therapeutic strategies. A paradigm deserving of critical attention is the integration of PROteolysis TArgeting Chimeras (PROTACs) into the therapeutic blueprint. Characterized by their unique mechanistic disposition to degrade rather than inhibit disease-culprit proteins, PROTACs herald a transformative phase in drug discovery (Mukerjee and Ghosh, 2023; Shaikh et al., 2023). Intriguingly, the molecular architecture of mangiferin and curcumin, illuminated by our findings, suggests a promising scaffold for PROTAC development. Their inherent binding affinities and preliminary insights into protein interactions imply a potential for tailoring these phytochemicals into specific degraders targeting the PI3K/Akt/mTOR pathway in ovarian cancer.

Complementing this is the evolution of nanotechnological delivery mechanisms. Embracing the promise of exosomal or liposomal delivery could redefine the therapeutic delivery landscape. Such advanced systems not only promise the encapsulation of these potentially revamped PROTAC entities but also ensure targeted, efficient, and biocompatible cellular delivery. By doing so, the therapeutic index is enhanced, and collateral cellular damage is minimized, addressing a long-standing challenge in oncological drug delivery. Yet, as the vistas of personalized medicine broaden, our journey mandates the incorporation of diverse patient data. Future endeavors should encompass a comprehensive analysis spanning genetic, epigenetic, and proteomic spectrums of ovarian tumours. This ensures that the derived therapies are not just potent but are tailored to individual patient profiles, maximizing therapeutic efficacy. In summation, while our research has charted untraversed territories, it is merely the dawn of an exhilarating journey. The amalgamation of mangiferin and curcumin attributes, potentially woven into the innovative realm of PROTACs and coupled with cutting-edge exosomal or liposomal delivery, heralds a potential renaissance in ovarian cancer therapeutics. A concerted interdisciplinary effort, sustained research rigor, and clinical validations will indubitably shape this promising horizon into a transformative reality.

## Data Availability

The original contributions presented in the study are included in the article/Supplementary Material, further inquiries can be directed to the corresponding authors.
